# Caudal migration and proliferation of renal progenitors regulates early nephron segment size in zebrafish

**DOI:** 10.1038/srep35647

**Published:** 2016-10-19

**Authors:** Richard W. Naylor, Rachel C. Dodd, Alan J. Davidson

**Affiliations:** 1 Department of Molecular Medicine and Pathology, University of Auckland, Auckland 1142, New Zealand

## Abstract

The nephron is the functional unit of the kidney and is divided into distinct proximal and distal segments. The factors determining nephron segment size are not fully understood. In zebrafish, the embryonic kidney has long been thought to differentiate *in situ* into two proximal tubule segments and two distal tubule segments (distal early; DE, and distal late; DL) with little involvement of cell movement. Here, we overturn this notion by performing lineage-labelling experiments that reveal extensive caudal movement of the proximal and DE segments and a concomitant compaction of the DL segment as it fuses with the cloaca. Laser-mediated severing of the tubule, such that the DE and DL are disconnected or that the DL and cloaca do not fuse, results in a reduction in tubule cell proliferation and significantly shortens the DE segment while the caudal movement of the DL is unaffected. These results suggest that the DL mechanically pulls the more proximal segments, thereby driving both their caudal extension and their proliferation. Together, these data provide new insights into early nephron morphogenesis and demonstrate the importance of cell movement and proliferation in determining initial nephron segment size.

The nephron is the smallest functional unit of the kidney and has a well-conserved structure and function amongst vertebrates. In general, the nephron is comprised of a blood filter (glomerulus) attached to a tubular epithelium with transporting and secretory functions. Filtrate generated in the glomerulus passes down the tubule and is sequentially modified as it passes through different tubule segments before being excreted from the body. The proximal tubule reabsorbs the bulk of the filtrate while the distal segments function more to ‘fine-tune’ the composition of the urine so that salt and acid-base levels in the body are kept within narrow limits.

Although our understanding of how the tubule segments arise during embryonic development is limited, important insights have been uncovered through the study of the anatomically simple zebrafish kidney^1^,^2^,^3^,^4^,^5^. The embryonic kidney (pronephros) in zebrafish is composed of two-nephrons on either side of the trunk that are fused rostrally at a common glomerulus as well as caudally at the cloaca^4^. The pronephric tubules are subdivided into two proximal segments (proximal convoluted tubule; PCT and proximal straight tubule; PST), and two distal segments (distal early; DE and distal late; DL)^2^. The PCT appears functionally equivalent to the proximal tubule of the mammalian kidney with a well-defined brush border, expression of a large cohort of solute transporters and high levels of endocytotic activity^2^,^6^. The PST, while also possessing a brush border, appears to function more in the transport of selective solutes, such as sulphate and magnesium^7^,^8^. In contrast to the proximal segments, the DE and DL segments express fewer transporter genes and are instead characterized by the expression of *slc12a1* (Na-K-Cl co-transporter) and *slc12a3* (Na-Cl co-transporter), respectively, indicating that these segments fine-tune sodium and chloride levels. Furthermore, as *slc12a1* is specifically expressed in the thick ascending limb (TAL) segment of the mammalian nephron^2^,^9^, it suggests that the DE and TAL may have a common evolutionary origin and thus share some functionality. The pronephric urine that is generated following transit through the proximal and distal segments leaves the embryo via the cloaca, which becomes fused during pronephric development to the caudalmost ends of the DL segments.

The pronephros arises from bilateral stripes of intermediate mesoderm towards the end of gastrulation^10^. Detailed expression analyses have shown that while early acting renal transcription factors encoded by *pax2a*, *pax8* and *hnf1b* are initially expressed throughout the intermediate mesoderm, by early somitogenesis the intermediate mesoderm can be subdivided into distinct rostral and caudal populations^11^,^12^. As development of the pronephros progresses, the rostral domain appears to expand while the caudal domain shortens. For instance, the *mecom* gene initially has an anterior expression boundary level with somite 6–7 but this gradually contracts until it is level with somite 14 at the 24 hours post-fertilization (hpf) stage^13^. While we initially interpreted these changes as reflecting dynamic spatiotemporal changes in gene expression, the fates of the cells in the rostral and caudal subdomains were not directly examined by lineage labelling experiments.

Considerable evidence indicates that the patterning of the intermediate mesoderm into rostral and caudal subdomains occurs in response to retinoic acid (RA) signalling. Zebrafish mutants defective in RA synthesis, or embryos treated with inhibitors of RA signalling, show a loss or reduction in the size of the rostral subdomain and a concomitant expansion in the size of the caudal subdomain^1^,^2^,^10^,^14^. These changes are associated with altered proximo-distal patterning of the tubule segments at later stages, as read-out by segment-specific transporter genes, with RA-deficient embryos showing reduced or absent proximal segments and expanded distal segments. However, the relationship between the early rostral/caudal subdomains and the later proximal/distal segments of the pronephros is unclear. While it is known that the pronephros has formed all of its tubule segments by 24 hpf, a detailed time-course of segment-specific transporter genes has not been performed. Furthermore, as RA can only affect pronephric patterning up to the 8-somite stage^10^, well before the full complement of nephron segments are detected with these markers, other non-RA pathways are likely to be involved in segment patterning. Recent studies have started to identify these factors, with the transcription factor genes *irx3b*, *sim1a*, *taz*, and *mecom* being implicated in determining the sizes of specific segments downstream of RA^11^,^13^,^15^,^16^. In addition, studies at later stages of pronephros development (>24 hpf) have shown that segment size is not static but instead highly dynamic. Once glomerular filtration commences the proximal segments undergo compaction in response to luminal flow, while the DE and DL segments elongate in response^17^. Thus, late-acting mechanisms exist to regulate segment size, which are independent of earlier-acting patterning factors such as RA.

In order for the pronephric urine to be excreted, the DL segment must fuse with the cloaca. As expression of *pax2a* extends around the tailbud in a horseshoe-like pattern, it is thought that DL progenitors are included in this domain and therefore they must be located close to the site of the cloaca. As a result, only a small amount of migration is assumed to be needed to fuse the caudal end of the DL segment with the cloaca^18^,^19^,^20^,^21^. This contrasts with mammals and amphibians, where the nephric duct lineage arises from the intermediate mesoderm in the upper trunk region and then migrates a considerable distance along the embryo to fuse with the cloaca^22^,^23^,^24^.

Here, we use gene expression analyses, fate mapping and cell ablations to examine the patterning and morphogenesis of the intermediate mesoderm during early pronephros development. We find that the intermediate mesoderm, rather than being a relatively static stripe of cells that differentiates *in situ*, shows dynamic cellular movements and active proliferation. DL progenitors are found to move caudally towards the cloaca and in doing so, the other tubule segments are ‘pulled’ down the embryo. This tensile force was found to influence the size of the DE segment, most likely via a mechanism involving stretch-activated proliferation.

## Results

### Expression of *hnf1ba* defines the intermediate mesoderm

**Figure 1:**
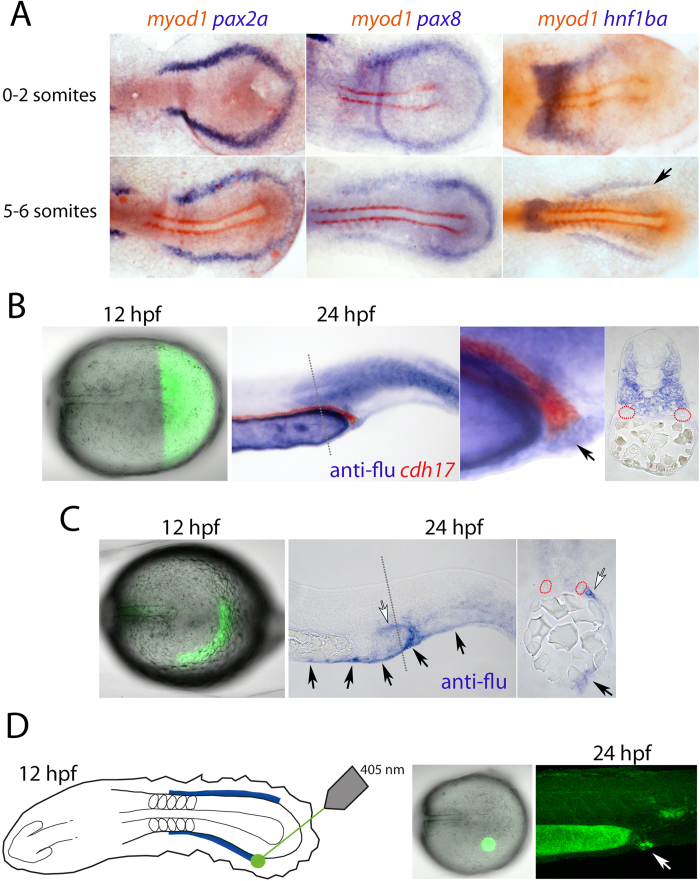
Lineage labelling of the intermediate mesoderm. (**A**) Panels show dorsal views of flat mounted embryos double stained for *pax2a*, *pax8* and *hnf1ba* (purple) and *myod1* (red) transcripts at the 0-2-somite and 5-6-somite stages. The caudalmost limit of the *hnf1ba* expression domain in the intermediate mesoderm is indicated with a black arrow. (**B**) Panels show the results of uncaging a lineage tracer in cells posterior to the end of the notochord (first panel) and the descendants of the labelled cells (purple) compared to the unlabelled *cdh17*-expressing kidney tubules (red; second and third panels, arrow in third panel labels cloaca cells). The fourth panel shows a cross-section, as indicated by the grey dotted line, of the embryo in panel two (red staining has been lost, but red dotted lines highlight the pronephric tubules). (**C**) Left panel shows lineage labelling of the ‘horseshoe’ domain at the 5-somite stage (fluorescence observed in the more rostral midline is background). Middle panel shows their contribution to non-renal cell types at 24 hpf by whole mount *in situ* hybridization. Labelled ventral fin cells are highlighted with black arrows and a population of mesenchyme lateral to the posterior pronephros is indicated with a white arrow, which is also highlighted in the cross-section view in the right panel. (**D**) Panels show lineage labelling of cloacal precursors (white arrow) located in a small cluster of cells near the end of the intermediate mesoderm. Images in (**B,C,D**) are shown in lateral view.

In previous studies we demonstrated that *hnf1b* is critical for the differentiation of the intermediate mesoderm into segmented pronephric tubules^1^. In the course of this work we noticed that transcripts for *hnf1ba* do not extend completely around the tailbud like that seen for the classic intermediate mesoderm markers *pax2a* and *pax8* (Fig. 1A). Instead, the expression domain of *hnf1ba* ends approximately level with the caudal limit of the notochord, while *pax2a*/*8*^+^ cells continue in a ‘horseshoe’-like pattern in the mesoderm layer (Fig. 1A and data not shown). To investigate the fates of the cells around the tailbud, we undertook a series of lineage-labelling experiments. Embryos were injected with a caged fluorescein dextran lineage tracer at the one-cell stage and the tracer was uncaged at the 5-somite stage. Initially, we labelled a large population of cells posterior to the end of the notochord and analysed these embryos at 24 hours post fertilization (hpf; Fig. 1B). We detected the uncaged tracer throughout the lower trunk somitic mesoderm, caudalmost blood cells and in the tail, consistent with the labelling of non-segmented paraxial mesoderm, the end of the blood stripes, and tailbud derivatives (n = 3). Cross-sections through these embryos revealed that the pronephric tubules were unlabelled, indicating that the *pax2a*/*8*^+^ cells around the tailbud do not correspond to tubule precursors. However, cloacal cells were found to be labelled by the tracer and co-staining for the pan-tubule marker *cdh17* showed fusion between unlabelled tubule and labelled cloaca (Fig. 1B). We next uncaged an arc of cells around the tailbud corresponding to half of the *pax2a*/*8*^+^ ‘horseshoe’ domain. These cells were found to populate the ventral midline of the yolk extension and tail (most probably fin mesenchyme), a population of mesenchyme lateral to the caudalmost pronephric tubules, epidermal cells around the end of the yolk extension and on the tail, and the cloaca (n = 9, Fig. 1C). To more precisely identify the location of cloacal progenitors, we uncaged cells in small regions within the ‘horseshoe’ domain, resulting in cloacal cells being localized to a region level with the caudal limit of the notochord (n = 3, Fig. 1D and data not shown). Based on these results we conclude that the intermediate mesoderm corresponds to the bilateral stripes of *hnf1b*^+^ cells (excluding the ‘horseshoe’ domain around the tailbud) while cloacal progenitors are localized at, or very near, the caudal limit of these stripes. While we did not specifically label the *pax2a*/*8*^+^ cells in the ‘horseshoe’ region, our labelling experiments are consistent with these cells giving rise to several non-renal lineages in the lower trunk and tail. Finally, we conclude that significant caudal and medial movement of both cloacal and tubule precursor cells must occur around the tailbud in order for these cells to end up, and fuse, at the ultimate site of the cloaca.

### Proximodistal patterning of the intermediate mesoderm

**Figure 2:**
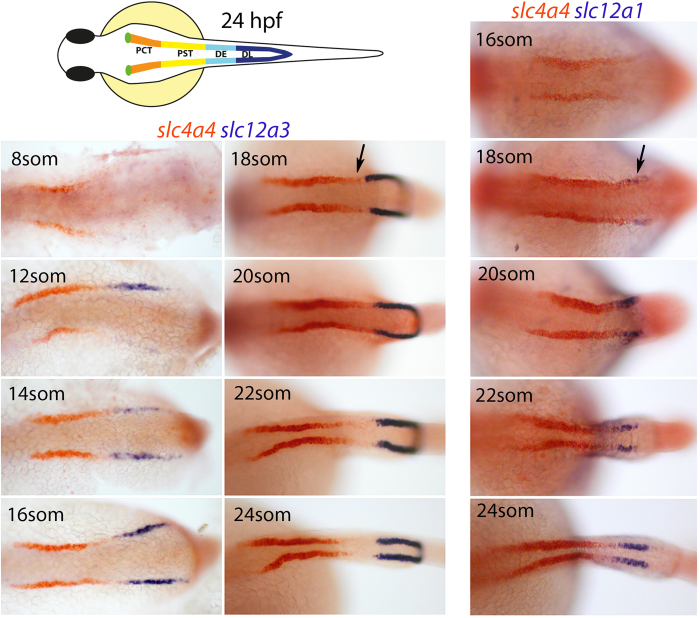
Expression analysis of segment formation in the pronephros. Panels show dorsal views of embryos double stained for *slc4a4* (red)/*slc12a1* (purple) and *slc4a4* (red)/*slc12a3* (purple) at the stages indicated. The position of initial *slc12a1* expression is highlighted with a black arrow and a schematic representation of the pronephros at 24 hpf is shown in the top left. Abbreviations; PCT, Proximal convoluted tubule; PST, Proximal Straight tubule; DE, Distal Early tubule; DL, Distal Late segment.

We next investigated the spatiotemporal patterning of the intermediate mesoderm into proximal and distal segments. To do this, we performed expression time-course analyses of *slc4a4*, a marker which eventually becomes restricted to the PCT/PST segments, *slc12a1*, a marker of the DE segment and *slc12a3*, a marker of the DL segment^2^,^25^. Of these genes, *slc4a4* transcripts initiate first, around the 8-somite stage, in the rostral subdomain of the intermediate mesoderm (Fig. 2). Expression of *slc12a3* appears slightly later, at the 12-somite stage, in the caudal subdomain of the intermediate mesoderm and abuts the *slc4a4*^*+*^ rostral subdomain. However, between the 18- to 24-somite stages, *slc4a4* transcripts become down-regulated in a small portion of the rostral subdomain, immediately adjacent to the boundary with *slc12a3*^+^ cells. Expression of *slc12a1* was found to initiate within this clearing, suggesting that the DE segment arises from rostral progenitors following their down-regulation of *slc4a4* (Fig. 2). Consistent with this, cells co-expressing *slc4a4* and *slc12a1* can be transiently detected at the 18-somite stage (Fig. S1A) and a sharp boundary between the expression domains of *slc12a1* and *slc12a3* is seen at the 20-somite stage (Fig. S1B). Together, these results suggest that by the 12-somite stage, the intermediate mesoderm is patterned into a rostral subdomain, containing presumptive PCT, PST, and DE progenitors and a caudal subdomain made up of DL segment progenitors. The appearance of the DE segment occurs later, at least by the 18-somite stage, based on the onset of *slc12a1* expression in rostral cells that down-regulate *slc4a4*.

### Rostral intermediate mesoderm progenitors move caudally and elongate

**Figure 3:**
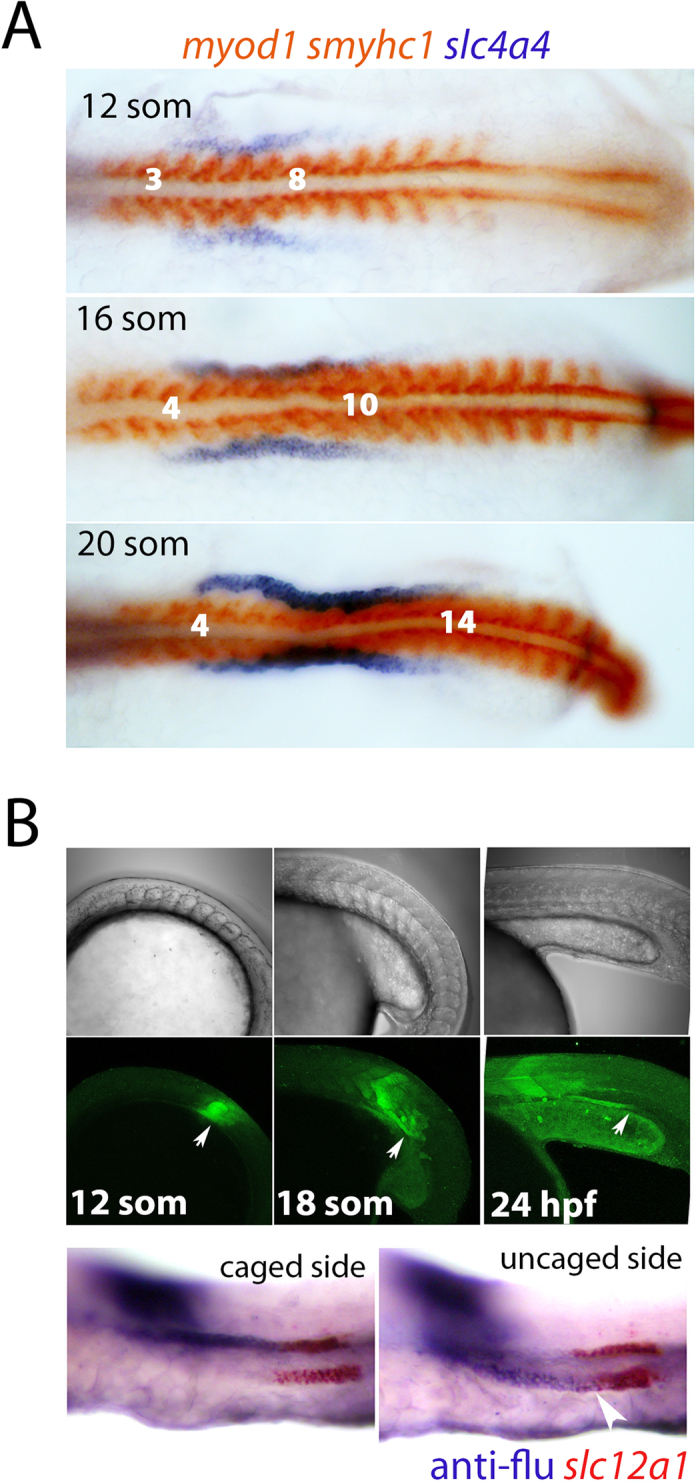
Lineage labelling of renal precursors. **(A**) Dorsal views of flat-mounted embryos that are double stained for *slc4a4* (purple) and *myod1*/*smyhc1* (red) are shown at the indicated stages. (**B**) Panels show uncaging of a lineage tracer in the intermediate mesoderm lateral to somite 8 at the 12-somite stage and tracking of these cells posteriorly (white arrows) up to the 24 hpf stage (upper panels show live brightfield images, middle panels show live fluorescent images). Lower panels show lateral views of the trunk from the same fate-mapped embryo double stained for the lineage tracer (‘anti-flu’; purple) and *slc12a1* (red).

We noticed during our time-course analyses that the *slc4a4*^+^ expression domain elongates caudally between the 12- to 20-somite stages, relative to the somites (Fig. 3A), consistent with previous observations of other rostrally-expressed genes^2^,^11^,^13^. While this caudal elongation of *slc4a4* may be the result of *de novo* gene expression in progressively more caudal portions of the intermediate mesoderm, it could also arise from rostral progenitors physically moving towards the tailbud. To distinguish between these possibilities we lineage-labelled a small cluster of intermediate mesoderm cells lateral to somite 8 at the 12-somite stage, corresponding to the caudal-most extent of the *slc4a4* expression domain at this time-point. Cells of somite 8 were also labelled as a reference for the starting location of the labelled intermediate mesoderm cells. Following uncaging, live embryos were analysed until the 24 hpf stage (n = 6, see Fig. 3B for one such example), revealing that the labelled intermediate mesoderm cells had moved caudally, forming a stretch of cells that reached to the level of somites 12–13. Double labelling for *slc12a1* and the uncaged fluorescein showed that the labelled cells contributed to the DE as well as more proximal portions of the tubule, most likely the PST (Fig. 3B, for close-up view see Fig. S2). These results provide good evidence to indicate that the expansion in rostral gene expression seen during intermediate mesoderm patterning is the result of the caudal movement of rostral progenitors. In addition, the fate-mapping experiments confirm that cells contributing to the DE can arise from the rostral domain.

### Compaction of the DL segment

**Figure 4:**
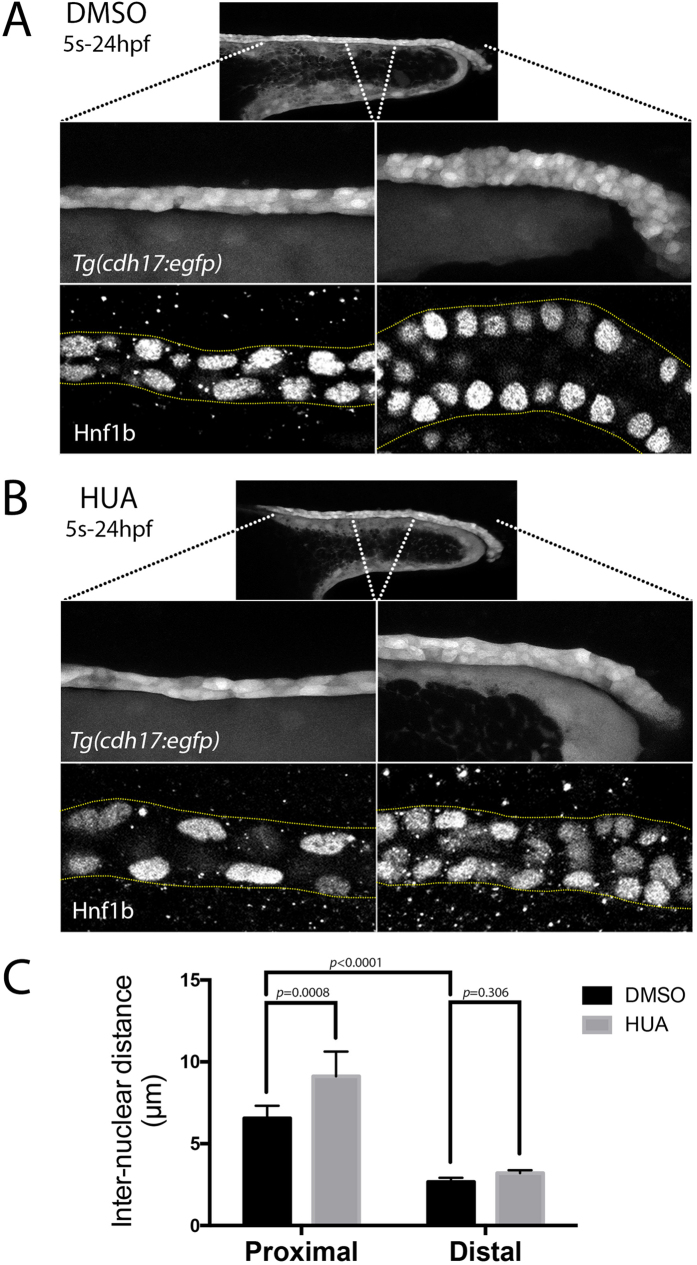
Live morphology of the pronephric tubule. (**A,B**) Top panels show a lateral view of the pronephric tubule in live *Tg(cdh17:egfp)* embryos at 24 hpf treated with either DMSO (**A**) or HUA (**B**). The two panels below show the same embryos with higher magnification views of the proximal (left) and distal (right) tubule. The bottom panels are single plane lateral views of Hnf1b stained pronephric nuclei in the proximal (left) and distal (right) tubule. (**C**) Histogram representing the inter-nuclear distances observed in the proximal and distal tubules of DMSO (control) and HUA treated embryos.

In support of the rostral progenitors moving caudally during the 12-somite to 24 hpf stages, we noticed that the *slc12a3*^+^ DL segment became more compacted during this time window (Fig. 2). This compaction was co-incident with the onset of migration of the distal-most end of the DL towards the cloaca, which begins between the 16- to 18-somite stages (Fig. S3). To better visualize changes in DL cell morphology we examined live *Tg(cdh17:egfp)* embryos, where the renal tubules are fluorescently labelled with green fluorescence protein (GFP)^1^, and found that by 24 hpf, the DL segment is made up of small rounded cells that are stacked 3–4 cells high (see Fig. S3 for still images taken from an 18-somite to 24 hpf time-course). By contrast, the neighbouring DE and PST regions comprise more narrow tubules made up of stretched epithelial cells consistent with being under mechanical tension (Fig. 4A). To quantitate these morphological differences, we immunostained embryos for Hnf1b at 24 hpf and measured the inter-nuclear distances of cells in the PST and DL segments. This analysis revealed that the average inter-nuclei distance for PST cells was ~2.5 times greater than DL cells (6.55 ± 0.76 μm and 2.68 ± 0.24 μm, respectively; n = 8; Fig. 4C), consistent with the DL being compacted relative to the PST. In further support of this, the PST cell nuclei are elongated along the anterior-posterior axis of the tubule while DL cell nuclei adopt a more rounded morphology (Fig. 4A). Taken together, these data support a model in which caudal movement and elongation of rostral (PCT, PST, DE) progenitors is associated with the migration and compaction of DL progenitors towards the cloaca.

### Blocking proliferation does not affect the caudal movement of rostral progenitors

**Figure 5:**
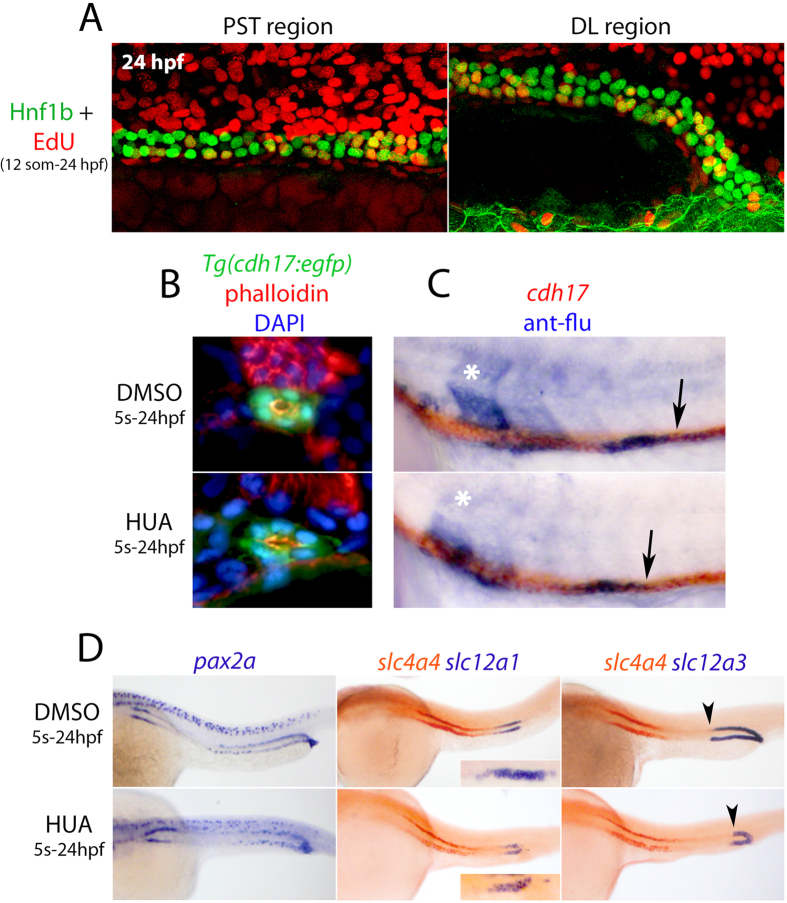
Effects of HUA treatment on tubulogenesis and caudal migration. (**A**) Left panel shows the PST region and the right panel shows the DL region of an embryo labelled with EdU (red) and Hnf1b (green) at the 24 hpf stage. (**B**) Transverse sections of *Tg(cdh17:egfp)* embryos treated with HUA and DMSO and stained with Phalloidin to label F-actin and DAPI to label nuclei. (**C**) Panels show lateral views of HUA and DMSO treated embryos double stained for *cdh17* and uncaged fluorescein (lineage tracer) that was labelled in the intermediate mesoderm lateral to somite 8 at the 12-somite stage. (**D**) Oblique lateral views of embryos treated with HUA and DMSO (control) and analysed for *pax2a*, *slc4a4*, *slc12a1* and *slc12a3* transcripts (black arrowheads indicate the anteriormost position of the DL segment).

The caudal movement of the rostral progenitors may be the result of cell proliferation occurring preferentially in PCT, PST, or DE progenitors. We first examined proliferation in the pronephros by labelling wild-type embryos with the thymidine analogue 5-ethynyl-2′-deoxyuridine (EdU) from the 12-somite stage onwards and then performed co-staining for Hnf1b at 24 hpf (Fig. S4). Widespread labelling of EdU was found along the tubule (data not shown) with quantitation in the PST and DL regions showing that ~40% and ~20% of PST and DL cells, respectively, had undergone division during this time window (n = 6, Fig. 5A and Fig. S4). We next tested whether cell division was necessary for the caudal movement of the rostral progenitors. To do this, we treated embryos with a mixture of hydroxyurea and aphidicolin (termed HUA) that effectively blocks cell division as previously described (see ref. 26 and Fig. S5). Treatment of *Tg(cdh17:egfp)* embryos from the 5-somite stage onwards with HUA reduced the width of the DL segment at 24 hpf (Fig. 4B), but did not grossly affect pronephric tubule formation or apical-basal polarity (Fig. 5B). Repeating the lineage labelling of rostral progenitors adjacent to somite 8 in the presence of HUA showed that inhibiting proliferation did not significantly reduce caudal migration (n = 8, Fig. 5C). To investigate the effect of inhibiting proliferation on tubule segment size we examined the expression of *slc4a4* (PCT, PST), *slc12a1* (DE), *slc12a3* (DL) and *pax2a* (DL and cloacal cells) in HUA-treated animals at 24 hpf (Fig. 5D). We found that while the PCT and PST segment lengths were similar in HUA-treated and control embryos and docking of the DL to the cloaca occurred normally, the DE segment and the DL segments were much smaller in all HUA-treated embryos (n = 35, Fig. 5D). Despite the length of the DL segment being reduced following HUA treatment, the inter-nuclear distance of DL cells was not significantly affected, suggesting that caudal compaction was able to occur normally, albeit with fewer cells (Fig. 4B,C). By contrast, proximal tubule cells displayed an increased stretched morphology in HUA-treated animals and inter-nuclear distance significantly increased in the PST region by ~1.4 fold compared to control animals (9.11 ± 1.52 μm vs 6.55 ± 0.76 μm; n = 8; Fig. 4C). We conclude from these results that cell division is unlikely to be a driving force for the caudal movement of rostral progenitors. Furthermore, when proliferation is blocked the smaller DL segment is still capable of undergoing caudal migration and compaction, with the more rostral progenitors becoming severely stretched.

### The DL segment drives caudal movement

**Figure 6:**
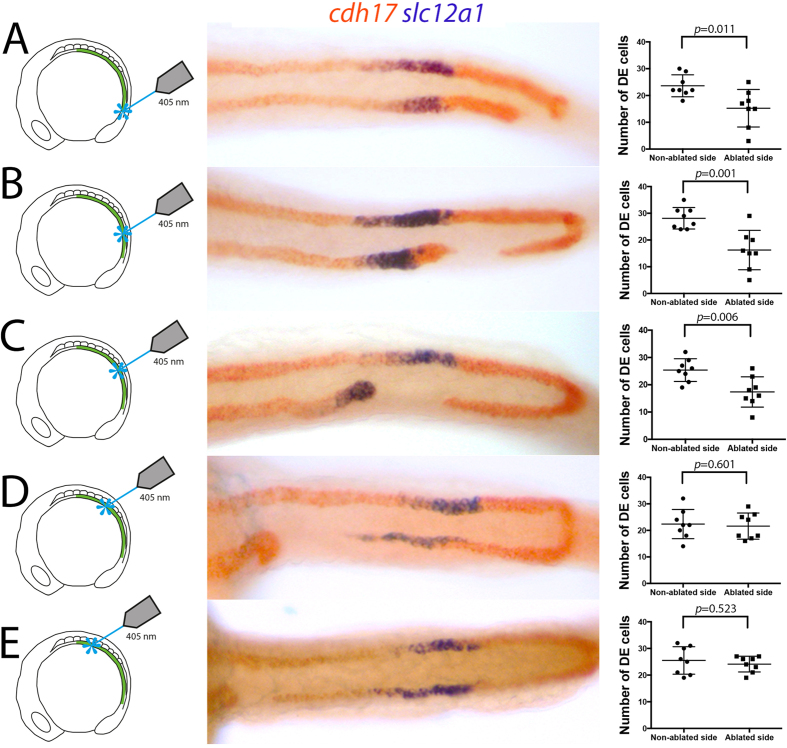
Laser ablation of the DL segment reduces DE segment size. **(A–E**) Dorsal views of the trunk of 24 hpf embryos that have undergone laser ablation of the intermediate mesoderm at the 12-somite stage at the indicated positions and double-stained for *cdh17* (red) and *slc12a1* (purple) transcripts. Manual cell counts of *slc12a1*^+^ cells are shown as dot-plots to the right.

Given that the DL segment undergoes compaction, we next examined whether this phenomenon was responsible for pulling the rostral progenitors caudally. We reasoned that because tubule epithelialization occurs between the 12- and 20-somite stages (ref. 27 and Fig. S6), compaction of the DL segment could generate mechanical tension along the tubule, via epithelial junction connections, and physically pull the rostral cells towards the cloaca. One prediction from this model is that rostral progenitors that are physically separated from DL progenitors should not move caudally. To test this, we used a laser to unilaterally ablate pronephric progenitors along the anterior-posterior axis of *Tg(cdh17:egfp)* embryos at the 12-somite stage (images of an embryo immediately pre- and post-ablation are shown in Fig. S7). The ablated embryos were then co-stained for the expression of *cdh17* and *slc12a1* at 24 hpf to determine the extent of DE cell migration (Fig. 6). We found that ablation of the caudal-most cells of the intermediate mesoderm (corresponding to the specialized tip cells that dock the DL to the cloaca) prevented cloacal fusion but did not significantly affect caudal migration (Fig. 6A). However, when DL cells were ablated in the middle of the segment, fewer DL cells remained attached to the DE segment and a greater reduction in the caudal movement of the DE segment was found (Fig. 6B). Laser ablation of the pronephros to isolate the DE segment entirely from the DL segment caused a loss of caudal migration (Fig. 6C). These results suggest that all DL cells have an intrinsic ability to migrate caudally, while the movement of the PCT, PST and DE segments is dependent on the ‘pulling’ action of the DL segment. In support of this, when ablations were performed within the PST and PCT segments, the DE segment on the ablated side appears drawn out and thin, consistent with it being dragged caudally by the DL (Fig. 6D,E).

### Laser ablation and LY294002 reduce tubule proliferation

**Figure 7:**
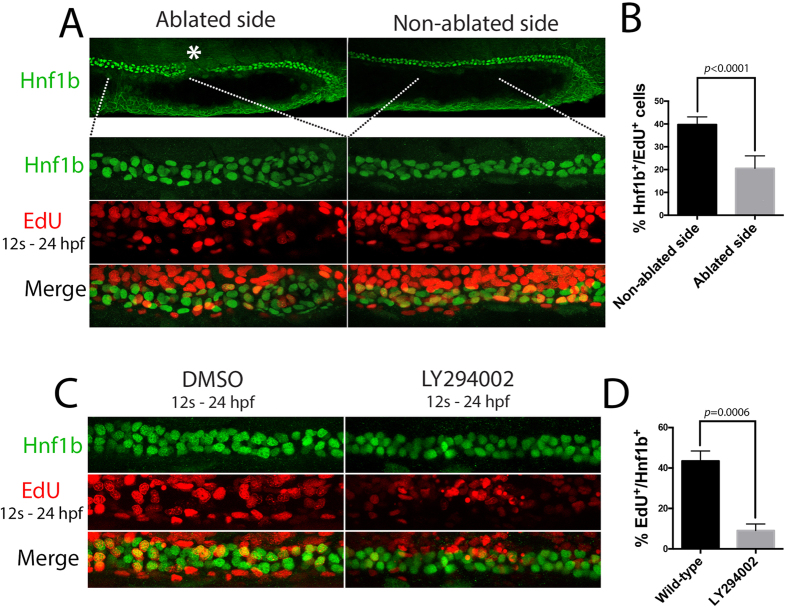
DL caudal compaction regulates proximal tubule proliferation and segment size. (**A**) Top panels show low magnification lateral views of the same embryo labelled with Hnf1b. Asterix denotes the site of ablation. Bottom panels show close-up views of the highlighted region of the ablated and contralateral control side of the embryo. (**B**) Histogram of the percentage of Hnf1b nuclei that are EdU^+^ in the control non-ablated side and ablated side of the tubule in 6 embryos. (**C**) Panels show lateral views of 24 hpf embryos treated with LY294002 or DMSO (control) and labelled with EdU from the 12-somite stage to 24 hpf. (**D**) Histogram of the percentage of Hnf1b nuclei that are EdU^+^ in control and LY294002 treated embryos.

Quantitation of *slc12a1*^+^ cells in embryos that had undergone ablation revealed that severing the tubule posterior to the DE segment reduced the number of DE cells while severing the tubule anterior to the DE segment had no effect (Fig. 6). These results suggest that DE cells may proliferate less when the tension provided by the caudally compacting DL segment is removed. To directly confirm this, the DE and DL segments were severed (as in Fig. 6C), labelled with EdU from the 12-somite stage onwards and immuno-stained for Hnf1b at 24 hpf (Fig. 7A). Quantitative analysis of these animals showed that the PST/DE region on the ablated side had ~2.0 fold less EdU^+^ tubule cells than the contralateral control side (n = 6, Fig. 7B). Given that at later stages (>29 hpf), tubular cells have been shown to proliferate in response to being stretched rostrally (as a mechanism to relieve tension), we speculate that the caudal compaction of the DL segment induces a similar response in the tubule in response to being stretched caudally^28^.

In the case of rostral stretching, the PI3-kinase pathway was found to be responsible for mediating stretch-induced proliferation^28^, prompting us to investigate the role of this pathway during DL migration/compaction. To do this, embryos were co-treated from the 12-somite stage onwards with LY294002, an inhibitor of PI3-kinase signalling, and EdU. Co-staining for Hnf1b and EdU showed a general reduction in proliferating cells along the entire tubule (data not shown). Quantitation of EdU labelling in the PST region (Fig. 7C) revealed a ~4 fold reduction in Hnf1b/EdU^+^ cells in LY294002-treated embryos compared to controls (Fig. 7D), while expression analysis showed that the DE and DL segments are shorter, similar to that seen in HUA-treated animals (Fig. S8). As the growth of all of the segments are affected by LY294002, including the DL segment, we conclude that the PI3-kinase pathway likely plays a more general role in mediating tubule cell growth between the 12-somite and 24 hpf stages, rather than specifically affecting stretch-induced proliferation.

## Discussion

Until now, studies of zebrafish pronephric tubule formation prior to 24 hpf have largely interpreted changes in segment size as patterning defects acting downstream of RA and have not addressed cellular parameters such as cell movement, stretch and proliferation. Our study demonstrates that the intermediate mesoderm does not statically differentiate into proximal and distal segments *in situ,* but is instead highly dynamic. Our marker analysis and fate mapping indicate that the RA-sensitive rostral progenitors likely comprise PCT, PST and DE precursors while the caudal progenitors correspond to the DL segment and undergo caudal migration and compaction. This caudally-directed movement may be important for positioning the DL segment and the closely associated cloaca progenitors at the site of the future cloaca where they fuse. Our analysis of the intermediate mesoderm at early somitogenesis stages showed that the intermediate mesoderm exists as bilateral stripes marked by *hnf1ba* transcripts, while the *pax2a*/*8*^+^ ‘horseshoe’ domain around the tailbud gives rise to mostly non-renal lineages and should not be considered *bona fide* intermediate mesoderm. As a result, the caudalmost end of the intermediate mesoderm must migrate some distance to arrive at the site of the future cloaca, ventral to the tailbud.

The caudal migratory characteristics of the DL, together with the expression of genes such as *emx1*, *aldh1a2* and the *ret* receptor and co-receptors, homologues of which are expressed by the nephric duct in mammals, birds, and amphibians^24^,^29^,^30^,^31^,^32^,^33^,^34^, suggests that the DL segment may be equivalent to the nephric duct of other vertebrates. However, expression of *slc12a3,* as well as *clcnk* and *kcnj1a.1*^35^, in the DL segment indicates that it also plays a role in solute reabsorption, unlike the nephric duct of birds and mammals. Given this, we suggest that the DL segment should be considered a tubule/nephric duct hybrid segment. While ‘pronephric duct’ identity has previously been conferred to the caudalmost region of the DL, this is largely based on the expression of a single gene, *gata3*, which is expressed by the nephric duct in mammals^36^. As *gata3* also controls nephric duct guidance^23^, it is possible that the *gata3*^+^ DL terminus reflects a subpopulation of specialised DL tip cells that are involved in cloacal docking (discussed further below).

Our results demonstrate that it is the caudally-directed movement of the DL segment that pulls more proximal progenitors towards the cloaca. As a consequence, any molecular markers of the rostral subdomain of the intermediate mesoderm (corresponding to PCT, PST, and DE precursors) will appear to elongate caudally and this is readily observed for genes such as *slc4a4* (as shown here) and *jagged-*2b^11^. Similarly, the progressive compaction of the caudal subdomain of the intermediate mesoderm (DL precursors) is also seen with caudal markers such as *slc12a3*, *mecom,* and *emx1*^3^,^11^,^13^. Because of these dynamic morphogenic movements, rather than dynamic changes in gene expression, tubule segmentation phenotypes should be interpreted cautiously. For instance, knockdown of *mecom*, a gene implicated in cell proliferation^37^, reduces the size of the DL segment and causes an expansion in the size of the PST segment and a caudal shift in the DE segment^13^. Rather than representing a patterning defect *per se*, this phenotype may instead be the result of less proliferation by DL precursors (giving a smaller DL segment) and the PST segment being stretched a greater distance along the trunk.

The molecular pathways governing the caudal movement and compaction of the DL segment are not well characterized. Studies in amphibians, chick and mice have shown that the caudal extension of the nephric duct occurs via a process of elongation, such that rostral portions of the duct remain in a fixed position while the caudal end extends towards the cloaca^22^,^23^,^30^,^31^,^32^,^34^. Recent studies in chick suggest that the FGF pathway promotes nephric duct migration^33^. Posteriorly shifting expression of *Fgf8* in the somitic mesoderm also prevents epithelialization of the tip cells of the duct and promotes elongation towards the caudally positioned cloaca^38^. Such a pathway may be conserved in zebrafish as *fgf8a* in the presomitic mesoderm shows a similar posterior shift in expression. In zebrafish, the DL segment reaches and fuses with the cloaca by the 22-somite stage^19^. Cells at the end of the DL segment extend numerous filopodia, consistent with the notion that there are specialized tip cells that interpret chemo-attractants released from the cloaca and guide docking^21^. When we ablated the tip region of the DL we observed a failure in cloacal docking, confirming the importance of the tip cells in coordinating DL-to-cloacal fusion. Surprisingly, the DL segment still underwent substantial caudal movement and compaction in these embryos, suggesting that all DL cells have an intrinsic ability to move. As such, tip cells may simply function to guide cloacal docking of the caudalmost end of the collectively migrating DL segment.

Our analysis of live cell morphology and inter-nuclear distances, together with tubule ablations that disconnect the DL segment from the other nephron segments, indicates that the DL segment moves caudally and exerts mechanical tension on the more proximal segments. We believe this accounts for the elongated/stretched appearance of the PST and DE segments compared to the DL segment at 24 hpf. This process is highly reminiscent of the tubule stretching seen later in pronephric development (from 29 hpf onwards), where the PST and DE segments are stretched in the opposite direction (rostrally) by compaction of the PCT segment^17^. Elegant live-cell analyses of this rostral stretching revealed that cells in the region of the DE and DL segments undergo stretch-activated proliferation via PI3-Kinase signalling and in response to fluid flow^28^. Although the details of this pathway remain unclear, downstream factors may include stretch-activated ion channels, inhibition of expression of cyclin dependent kinase inhibitors, and the Taz transcription factor^16^,^39^,^40^,^41^,^42^. We find that DE segment size is affected by blocking cell division with HUA or LY294002 (an inhibitor of the PI3-Kinase pathway) and physically separating the DE segment from the DL segment reduced proliferation. These data support a model in which the caudal compaction of the DL stretches the proximal pronephros and induces proliferation. However, our ablation experiments suggest that caudal migration of the DL is not sufficient by itself to induce DE proliferation, as ablating the tip cells, which only slightly reduces DL migration, had a similar effect on DE cell reduction as ablations that more severely affected DL migration. To account for this, we speculate that attachment of the DL segment to the cloaca acts as an anchor for the tubule so that the compaction force of the DL segment can be physical transmitted along the tubule, thereby inducing stretch-activated proliferation in more proximal segments. Such a pathway would provide a homeostatic mechanism to ensure that the tubule lengthens in-step with progressive axial lengthening, such as that arising from convergence-extension movements and growth^43^. The finding that this process reverses direction once flow initiates, with the proximal segments ‘pulling’ the more distal segments rostrally, highlights how nephrogenesis involves a dynamic see-sawing of inter-tubular forces that induce corresponding changes in segment proliferation and cellular morphology. The challenge for the future will be to determine how these forces shape the mammalian nephron, which is much more difficult to study *in vivo*.

In summary, our data show that the zebrafish intermediate mesoderm is initially subdivided into a proximal domain (which gives rise to the PCT/PST/DE segments) and a distal domain (which forms the DL segment). The DL segment migrates to the cloaca and compacts, and in the process, pulls the proximal segments causing them to elongate and proliferate in response to stretch. This process occurs after the intermediate mesoderm is patterned in response to RA (which occurs prior to the 8-somite stage) and highlights the need to evaluate cell movement and proliferation when interpreting tubule segmentation phenotypes in zebrafish.

## Concise Methods

### Zebrafish husbandry

Zebrafish embryos were maintained and staged according to established protocols^44^. Zebrafish experiments were performed in accordance with protocol 001343 approved by the University of Auckland Animal Ethics Committee. Paired matings of wild-type *Tübingen* (*Tg*) or transgenic *Tg(cdh17:egfp)* adults were carried out in order to collect embryos used to perform the experiments described.

### Whole mount *in situ* hybridization and antibody staining

Whole mount *in situ* hybridization was performed using protocols previously described^45^. Digoxigenin and Fluorescein anti-sense riboprobes were synthesized using T7/T3/SP6 RNA polymerase transcription kits (Roche Diagnostics) from plasmids used previously^2^. For double *in situ* hybridizations, alternative Dig- or Flu- riboprobes were used and alkaline phosphatase was inactivated by two 15 minute 100 mM Glycine (pH 2.2) treatments. To perform whole mount antibody staining, fixed embryos were washed twice in PBS containing 0.05% Tween20 (PBST), then placed in PBS containing 0.5% Triton X100 for 20 minutes to permeabilise the embryo. Embryos were then washed twice in TBS containing 0.05% Triton X100 (TBST) and blocked in TBST containing 3% BSA and 5% Goat serum for at least one hour. The Hnf1b primary antibody (SigmaAlrich #HPA002083, 1:1000) and β-catenin primary antibody (SantaCruz Ab #7199, 1:250) were added overnight at 4 °C. Embryos were washed four times in PBST before Goat anti-Rabbit IgG DyLight488 conjugated secondary antibody (Abcam #96899) was added at 1:500 dilution. Embryos were incubated in the secondary antibody for 2 hours at room temperature, washed twice in PBST then imaged on a FV1000 LiveCell confocal microscope (Olympus) where the red and green channels were imaged sequentially.

### Lineage labelling

2 μg/μl of dextran DMNB-caged fluorescein biotin 10,000 MW (Invitrogen Tomoko Obara NIH grant# R01 DK078209-04S1) was injected into embryos at the one-cell stage. Injected embryos were grown to the 12-somite stage, manually de-chorionated and placed into a well on a petri dish containing a bed of 1% agarose. Embryos were oriented dorsal side up and un-caging was performed by selecting a region encompassing somite 8 and the intermediate mesoderm lateral to this somite for a 10 second exposure to a 405 nm diode laser using a Sim Scanner on an Olympus FluoView FV1000 confocal laser scanning microscope. Un-caged embryos were placed in fresh E3 embryo medium and grown to 24 hpf. To detect un-caged fluorescein, the *in situ* hybridization protocol was followed and anti-fluorescein fragments (Roche Diagnostics) were used to label the un-caged fluorescein.

### Drug treatments

Embryos were de-chorionated and exposed to various drugs at time-points outlined in the text. Hydroxyurea (SigmaAldrich #H8627) was dissolved to a 1 M stock solution in water. Aphidicolin (SigmaAldrich #A0781) was dissolved to a 10 mM stock solution with DMSO. The combined Hydroxyurea (20 mM) and Aphidicolin (150 μM) solution (termed HUA in the text) was made up in E3 embryo medium. LY294002 (SigmaAldrich #L9908) was dissolved in DMSO to a stock concentration of 10 mM and diluted in E3 media to a working concentration of 30 μM. All control experiments used the highest equivalent vehicle concentration.

### Laser ablation

Our laser ablation protocol was similar to that described previously^46^. *Tg(cdh17:egfp)* embryos at the 12-somite stage were manually de-chorionated and placed into a well on a petri dish containing a bed of 1% agarose. Embryos were oriented dorsal side up and GFP-labelled intermediate mesoderm was photo-ablated using a 60X water-dipping objective. We found a 5 minutes exposure to a 405 nm diode laser at maximum intensity with a 12.5 μs/pixel dwell time was required to enable severance of the tubule, when observed later at 24 hpf (shorter time-points were tested but did not consistently sever the tubule). The specific target region corresponding to a circular area with a diameter of 25 μm was selected using a Sim Scanner on an Olympus FluoView FV1000 confocal laser-scanning microscope. Subsequent to photo-ablation, embryos were transferred to fresh E3 embryo medium and grown to 24 hpf for further analysis.

### EdU labelling

For exposure to the EdU label, *Tg(cdh17:egfp)* embryos were injected into the yolk with 0.5 μg/ml EdU (SantaCruz #284628). Embryos were left in E3 embryo medium for the periods of development outlined in the text (we found incubating embryos in E3 media containing EdU only permitted superficial EdU incorporation at post gastrula stages). Embryos were fixed at the desired stage and then incorporated EdU was detected using a Click-iT EdU Alexa Fluor 594 kit (Life Technologies).

### Cryosectioning and Phalloidin staining

Embryos were fixed in 4% PFA overnight at 4 °C then washed twice in PBST. Embryos were embedded in a 1% agarose block in a cryo-mould. After solidification for an hour at room temperature, the agarose block was placed in 30% sucrose in PBS and left for infiltration overnight at 4 °C. Once the block had sunk in sucrose, it was flash frozen on dry ice before being stored at −20 °C for cryo-sectioning. Sectioning was performed on a cryostat machine and 10 μm slices were collected. For Phalloidin staining, sections were washed twice in TBST then exposed to Phalloidin Dylight 594 (Abcam #21836, 1:3000 dilution) for 30 minutes in the dark at room temperature. Sections were washed twice with TBST and mounted in Prolong Gold (Life Technologies). For embryos stained for lineage tracer, cryo-sectioning was performed after labelling and sections were mounted under a cover slip with Entellan (SigmaAldrich).

### Statistical analysis

All experiments were performed at least three times and statistical analysis used unpaired t-tests with significance set a 95% confidence limits (*p* > 0.05). For the counting of inter-nuclear distances shown in Fig. 4, 20 adjacent Hnf1b^+^ nuclei were used for measurement in the proximal tubule (from the start of the yolk extension) and the distal tubule (immediately adjacent to the cloaca). For the counting of EdU^+^ and Hnf1b^+^ nuclei shown in Fig. 7A and Supplementary Fig. 4, nuclei in a 100 μm region of the proximal tubule and the distal tubule were counted. For the counting of EdU^+^ and Hnf1b^+^ nuclei shown in Fig. 7B, nuclei in a 100 μm region of the proximal tubule immediately anterior to the site of ablation were counted.

## Additional Information

**How to cite this article**: Naylor, R. W. *et al*. Caudal migration and proliferation of renal progenitors regulates early nephron segment size in zebrafish. *Sci. Rep.* **6**, 35647; doi: 10.1038/srep35647 (2016).

## Supplementary Material

Supplementary Information
