# Supplementary material for: Caudal migration and proliferation of renal progenitors regulates early nephron segment size in zebrafish
Source: Sci Rep. 2016 Oct 19;6:35647. doi: 10.1038/srep35647 (PMC5069491; doi:10.1038/srep35647)
Supplement: Supplementary Information [file srep35647-s1.pdf]

Caudal migration and proliferation of renal progenitors regulates early nephron segment size in zebrafish

Richard W. Naylor, Rachel C. Dodd and Alan J. Davidson*

Department of Molecular Medicine and Pathology, University of Auckland, Auckland 1142, New Zealand

* Corresponding author (a.davidson@auckland.ac.nz)

Richard W. Naylor: r.naylor@auckland.ac.nz, +64 022-062-8821

Rachel C. Dodd: r.dodd@auckland.ac.nz, +64 021-177-5071

Alan J. Davidson: a.davidson@auckland.ac.nz, +64 021-0811-7615

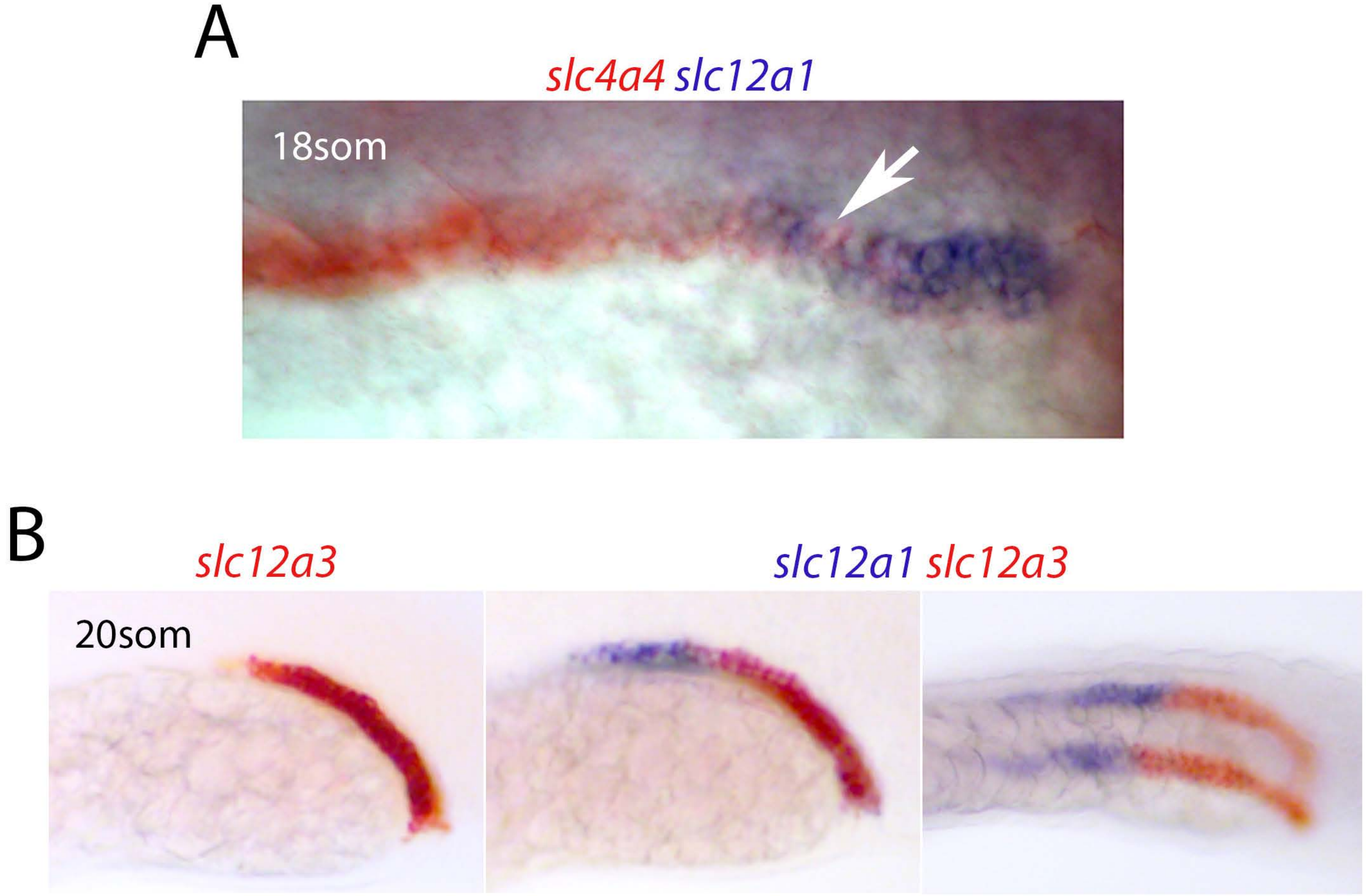

Supplemental Figure 1: A) Expression of *slc4a4/slc12a1* at the 18-somite stage. White arrow highlights mixing of these two populations. B) Left panel shows a lateral view of 20-somite stage embryo stained for *slc12a3* in red. Middle and right panels show the same embryo but also with *slc12a1* staining in purple with the middle panel being a lateral view and the right panel an oblique view.

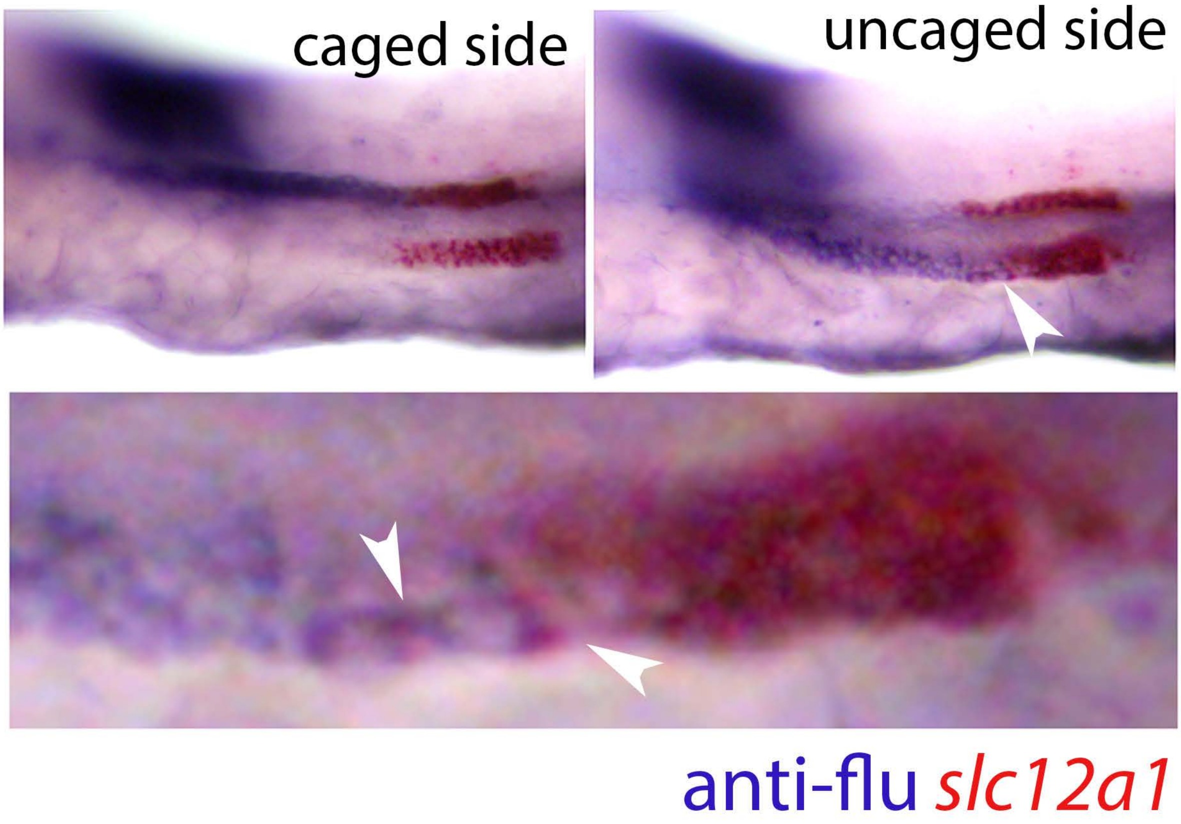

Supplemental Figure 2: Bottom panel shows close up view of uncaged cells that have migrated from lateral to somite 8 at the 10-somite stage to being lateral to somites 12-13 (equivalent to the DE segment) at the 24 hpf stage. Arrow heads in the bottom panel identify doubly labelled cells in the anterior domain of the DE segment.

Tg(cdh17:egfp)

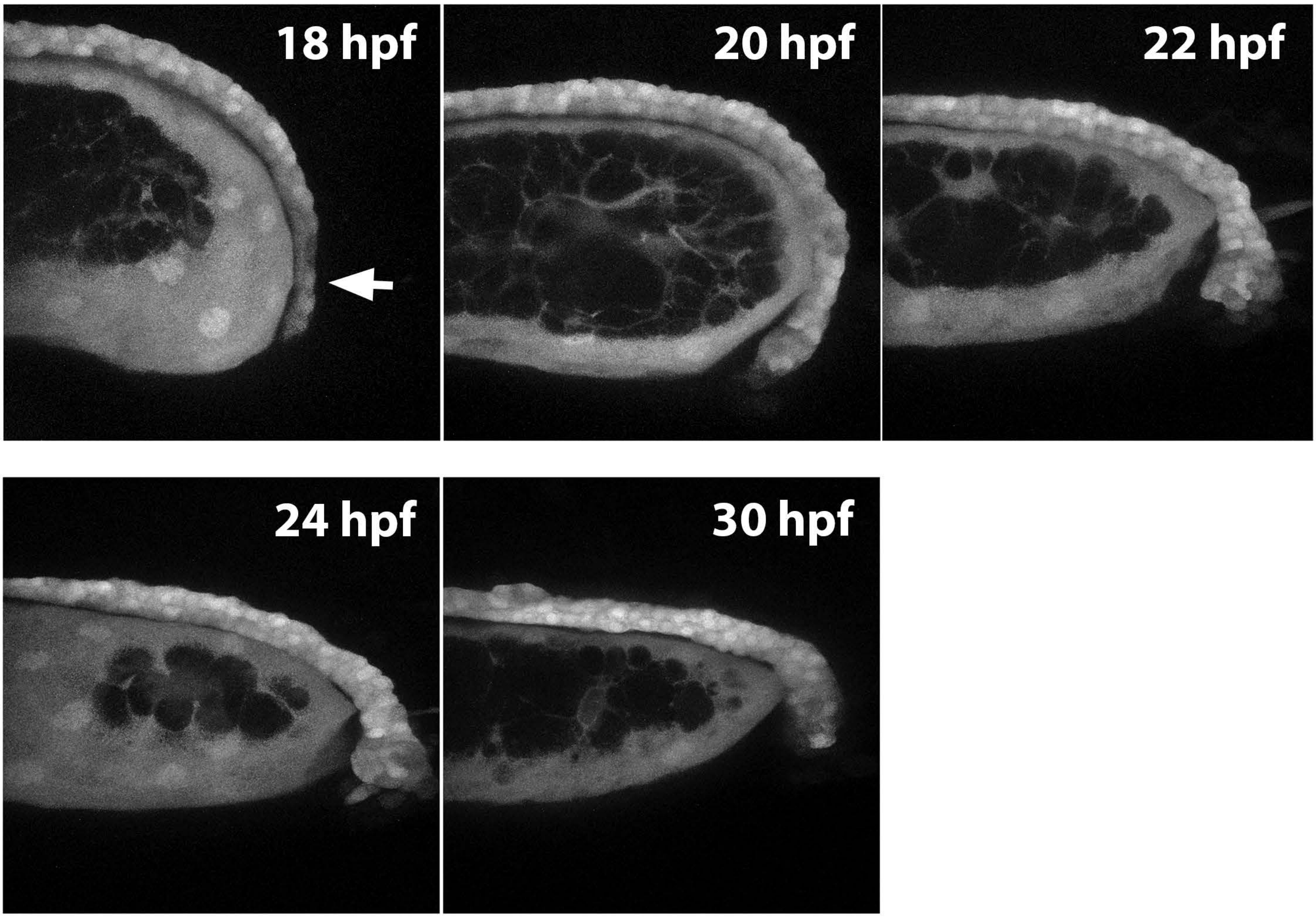

Supplemental Figure 3: Panels show stage series live capture of the same *Tg(cdh17:egfp)* embryo between 18 hpf and 30 hpf. White arrow in 18 hpf panel highlights the extending distal pronephros prior to caudal migration and cloacal fusion.

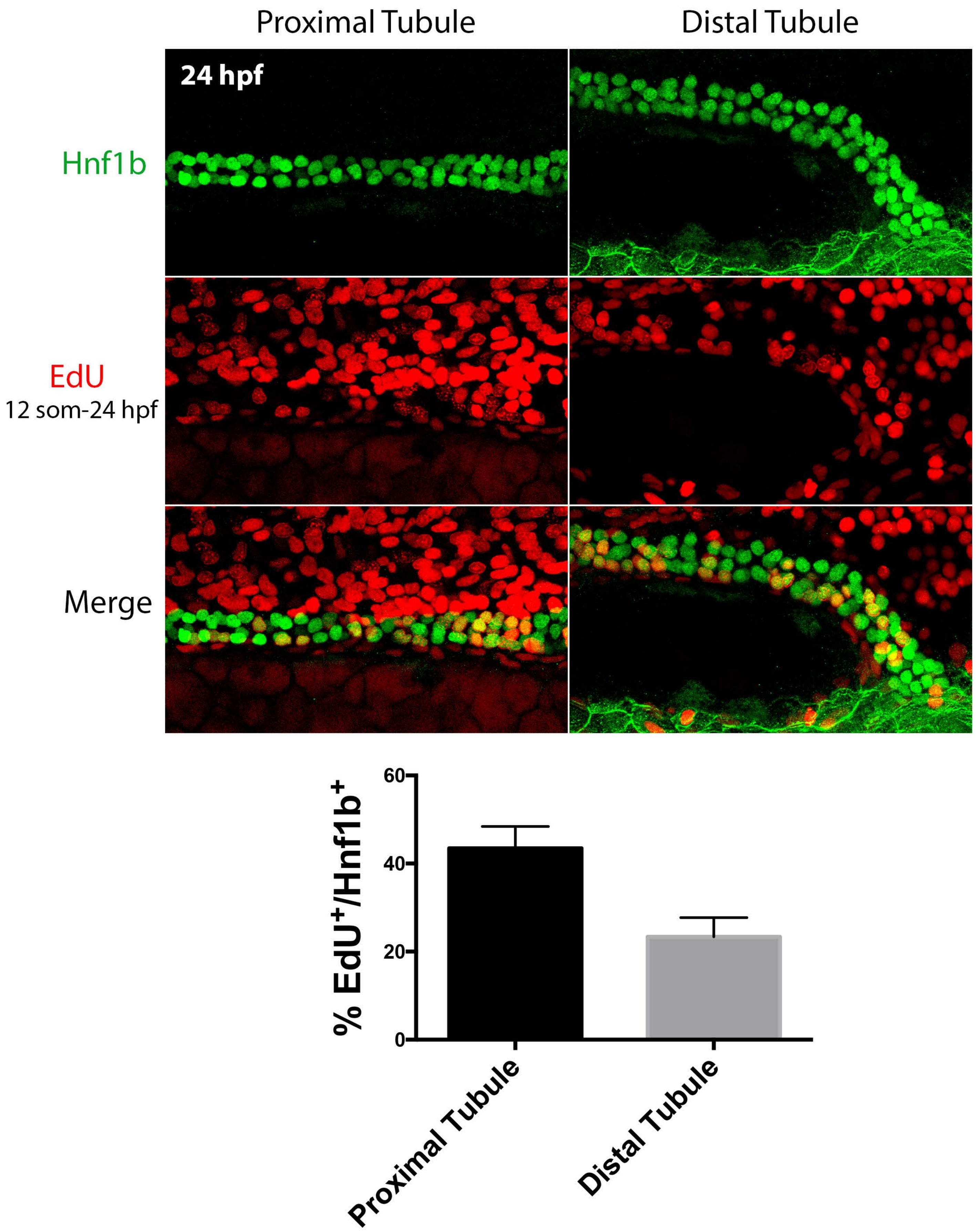

Supplementary Figure 4 Top panels show Hnf1b (green) and EdU (red) in the proximal tubule region (left) and distal tubule (right). Histogram at the bottom shows the % of Hnf1b nuclei that are labelled for EdU in the proximal tubule and distal tubule.

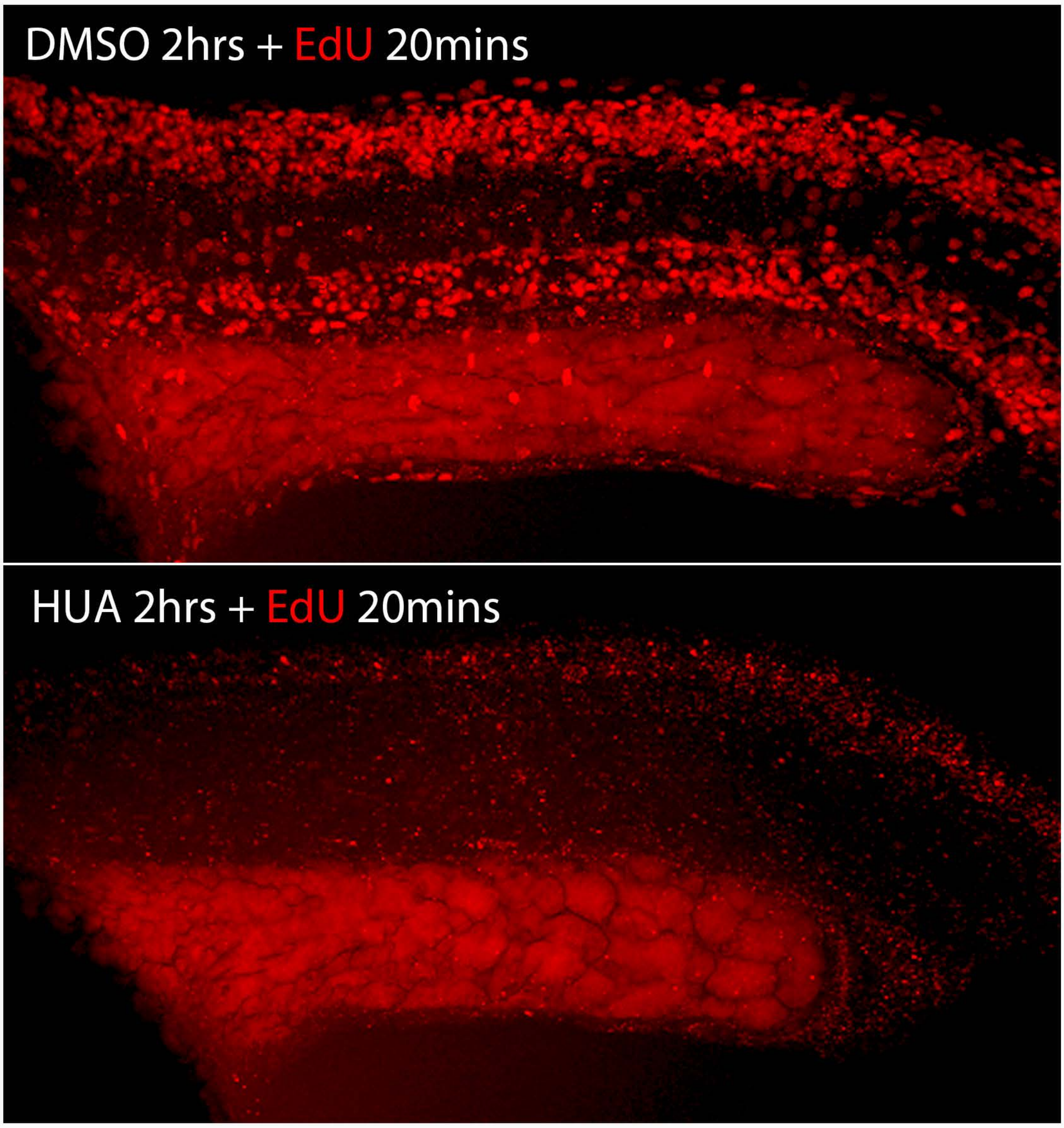

Supplemental Figure 5: EdU incorporation in HUA and DMSO (control) treated embryos. Panels show lateral views of embryos labelled with EdU (red) for 20 minutes after a two hour treatment with DMSO (control) or HUA from the 22-somite stage.

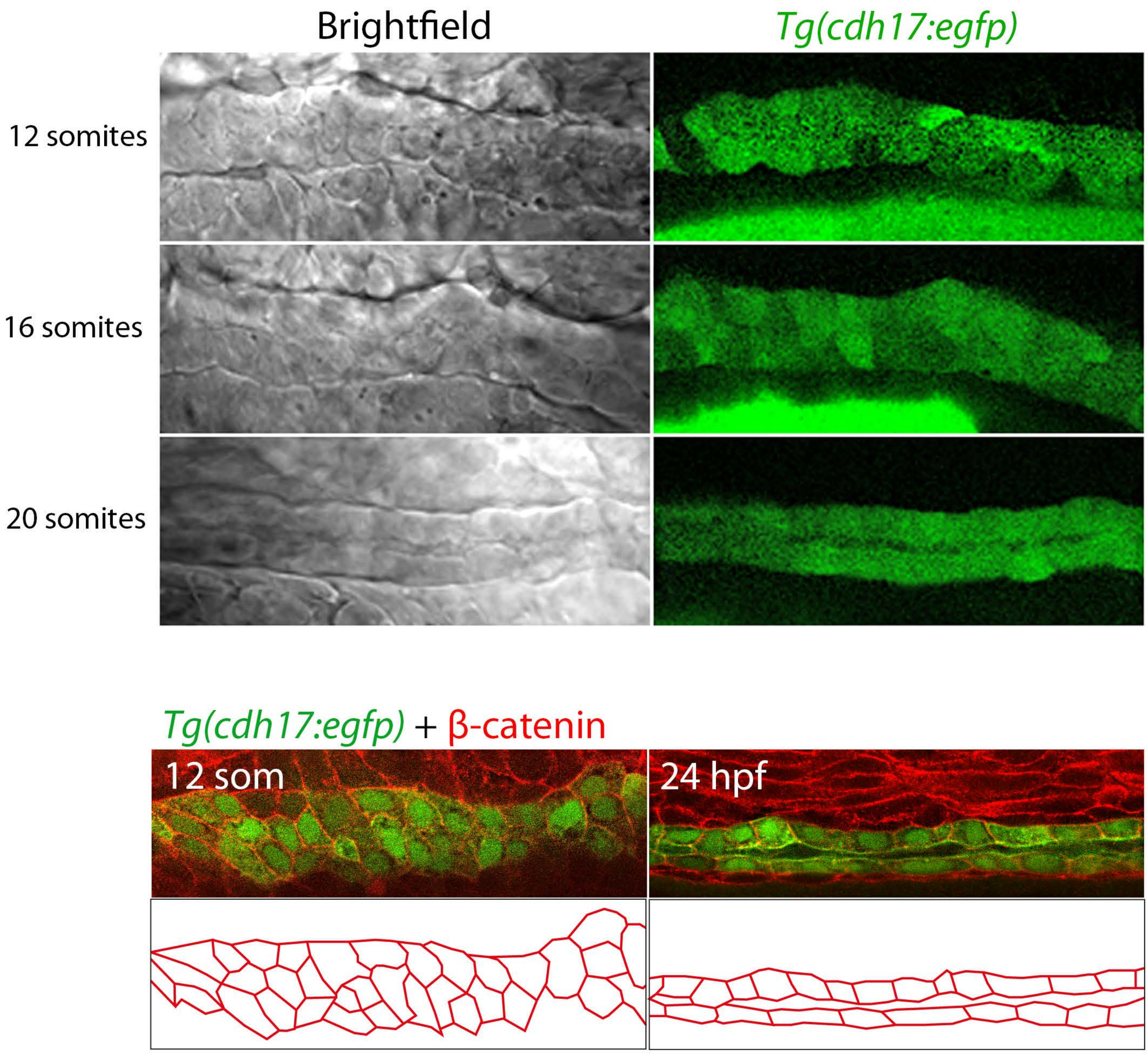

Supplemental Figure 6: Tubulogenesis in the pronephros. Top Panels show brightfield and fluorescence images of the intermediate mesoderm in *Tg(cdh17:egfp)* embryos. Bottom panels show lateral views of *Tg(cdh17:egfp)* embryos stained for β-catenin at the 12-somite and 20-somite stages (upper) with cell outline tracings (lower).

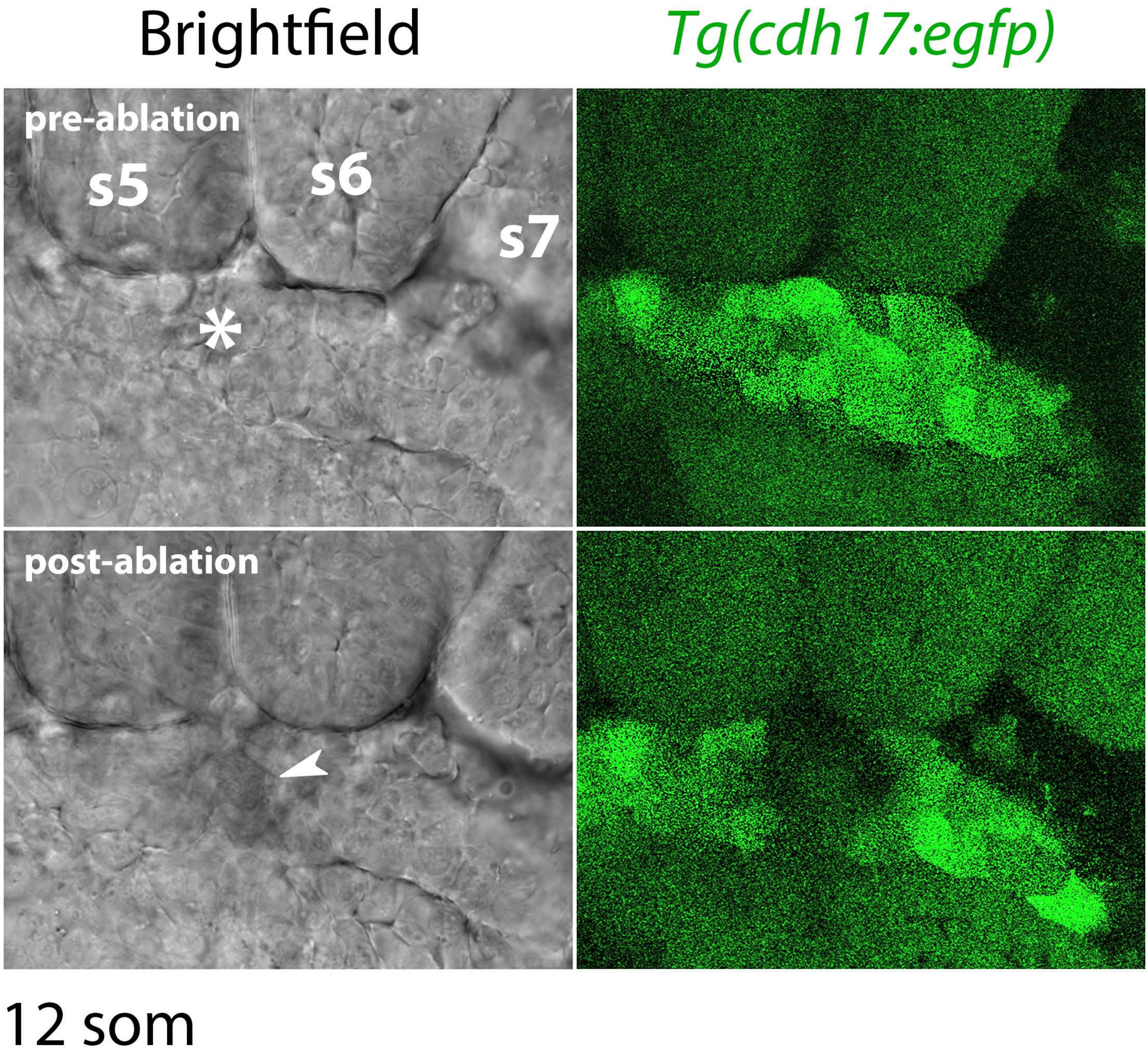

Supplemental Figure 7: Laser ablation of the tubule. Panels show close-up images of *Tg(cdh17:egfp)* embryos under brightfield and fluorescence microscopy at the 12-somite stage where 405nm wavelength light on a confocal microscope has ablated the intermediate mesoderm (asterisk marks target site for ablation, white arrowhead highlights the target site immediately post-ablation).

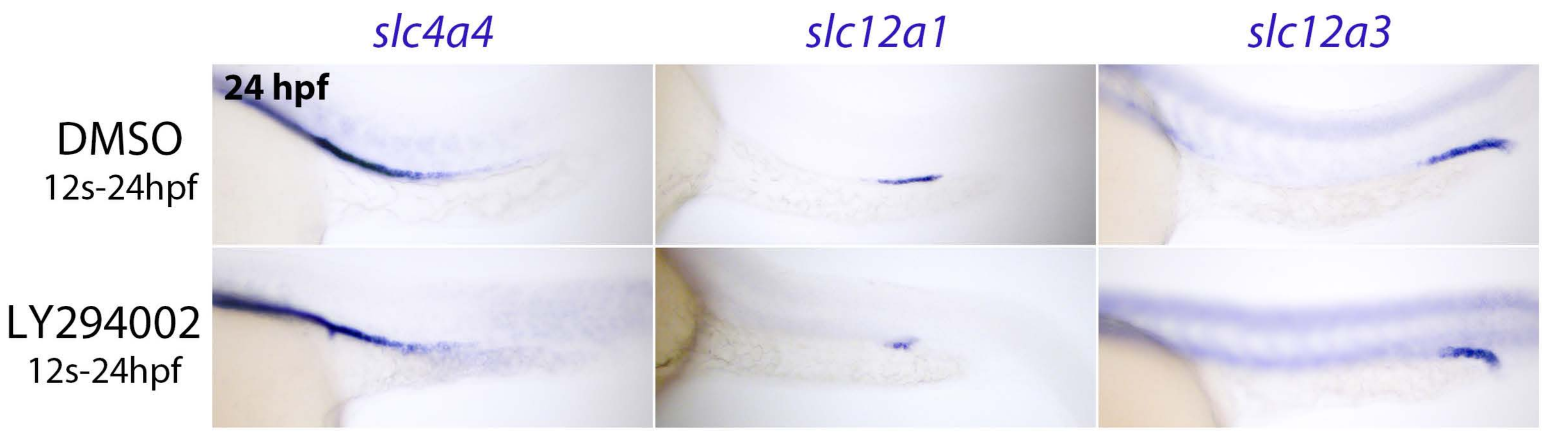

Supplemental Figure 8: Panels show lateral views of 24 hpf stage embryos labelled for *slc4a4*, *slc12a1* and *slc12a3* in control (DMSO) and LY294002 treated embryos.
